# Domestic

**Published:** 1841-03-27

**Authors:** 


### DOMESTIC.

*Traumatic Tetanus, successfully treated by Tobacco Enemas.* By B. E. Bowen, M. D.—On the evening of Friday, the 19th inst., I was requested to visit Miss C., aged twenty years, whom the messenger informed me had just been taken with cramp fits, as he expressed it, and desired me to lose no time in getting there. On my arrival, I learned that my patient had been for two or three days experiencing great pain in the right wrist, and on further inquiry I ascertained that near two years had elapsed since she had received a sprain of that wrist by the upsetting of a waggon, which at the time caused no great inconvenience, but at times since she had felt uneasiness and pain at the lower portion of the ulna. For the last three weeks the pain had been pretty constant, but not so severe until the last three days. Over the lower head of the ulna there was much tenderness, some degree of redness, and very little tumefaction.

Her history satisfied me that she had been to a considerable extent the subject of nervous irritation, as also of more or less dysmenorrhœa. The pain in the wrist had become so severe during the afternoon preceding my visit, that she had consulted a physician, who ordered the application of a blister, which had been on but a short time when I arrived. The appearances presented on my approaching the bedside, were redness of the face, general increased heat, eyes looking red and frequent escape of tears, pulse somewhat increased in force and frequency. In a very few moments a return of the spasm took place, which lasted from three to five minutes, characterized by great rigidity of the muscles, the body bending back, with a flexed condition of the forearms, fingers, and toes. As the spasm went off she would exert herself powerfully, making strong and quick inspirations, and manifesting great distress and consequent exhaustion. This relaxation of the muscles was of short duration, when a return of the spasm, perhaps still more severe, would take place. After witnessing several alternations as above described, I embraced the first opportunity afforded by the subsidence of spasm, and drew blood to the amount of twenty ounces. Deglutition was somewhat difficult, but I succeeded in the administration of calomel and opium, twenty grains of the former and six of the latter. During the intervals between the spasms the powers of sensation and thought were unimpaired, and the patient appeared conscious of what took place, and would complain of excessive pain in the wrist. Neither the bleeding nor the medicine procured any sensible relief. Repeated the opium every two hours in increased doses. Examined the wrist; vesication having taken place, removed the cuticle, and ordered the application of bread and milk poultice, with the addition of pulvis opii over the blister.

*Twelve o'clock, night.* Obliged to leave my patient. Ordered, at the expiration of two hours, opium and calomel, with ipecac. sufficient to nauseate the stomach.

*Saturday, 9 o'clock, A. M.*, returned to my patient. Ascertained that the spasms had ceased after taking the opium, cal. and ipecac., which was followed by perspiration and two or three hours' sleep. The spasms had now returned in a greater degree of severity than I had yet witnessed, with evident *trismus*, which continued somewhat longer than the general contraction of the muscles of the body and limbs, causing great suffering of the patient, as was evinced by her putting her fingers to the jaws and muscles of the neck. As soon as the jaws could be separated, opium and calomel twenty grains each were given, and in a short time followed by castor oil and spts. terebinth. Complained of uneasiness at the stomach, which was followed by vomiting the oil and turpentine. Spasms growing worse, causing great curvature of the body, bending it backwards in the form of an arch.

At this stage of the case, 12 o'clock, M., and about eighteen hours from the commencement of the spasms, I resolved on trying the tobacco enema, for which purpose about the third part of a threepenny paper of cut tobacco to one pint of boiling water was ordered, and of this infusion take the one-third for an enema. Five minutes having elapsed, patient begins to be much prostrated, coolness of the surface, and profuse perspiration breaking out; great relaxation of all the muscles, and jaws becoming loosed. In twenty minutes muscles very much relaxed; a feeling of extreme nausea with some vomiting. A great change is perceptible in the countenance; from fulness and redness, it has now become pale and contracted, and the whole body and limbs covered with perspiration. One hour and fifteen minutes having passed since the tobacco was given, more redness and heat of surface became apparent; pulse increasing in force and frequency, with some twitching of the eyelids, and a starting of tears. Spasms return, though not as violent. Two or three follow in quick succession. Repeat the tobacco enema, same quantity as before, which is followed by similar effect. Now obliged to leave my patient a few hours, and ordered a repetition of the enema every hour.

*Seven o'clock, P. M.*—She has taken the tobacco six times. No return of spasms unless the enema was omitted too long. I find that the tobacco is beginning to have a more specific effect; sickness and prostration more severe, with an increase of the vomiting. I also consider the tendency to spasm as being much diminished. Reduce the strength of the enema, and give the same at intervals of two hours, unless sooner required by the condition of the patient. The bowels have moved twice. She complains less of the wrist. Repeat the poultice every four hours, covering the same with moistened, cut tobacco.

*Saturday*, 6 *o'clock*, *A. M.*—Patient got some rest during the night. Considerable feverish action; mouth and tongue dry; complains of distress at the stomach. The jaws were set once during the night, but gave way after the administration of the tobacco. Give the *pulvis antimonialis* once in four hours, alternating with *spts. nit. dulcis*, and drinks of slippery elm, &c.

*Six o'clock*, *P. M.*—Has had no spasm during the day, and has not taken any of the *weed*. Some tendency to twitching of the muscles when she falls asleep. Give four grs. of the pulvis Doveri, combined with camphor, once in four hours.

*Monday*, 7 *o'clock*, *A. M.*—Rested well during the night; tongue moist, and all appearances highly favourable.

*Tuesday*.—She has been dressed, and is sitting up. Feels no pain in the wrist, and complains of nothing except feeling somewhat weak.

*Thursday*.—I consider my patient cured, her appetite having returned, and the wrist being free from pain.

*Mexico*, (*Oswego Co.*,) *N. Y.*, *Feb.* 27, 1841.

*Bost. Med. and Surg. Journ.*

---

*Weekly Report of Interments in the City and County of New York*, *from the* 13*th to the* 20*th of March*, 1841.—Diseases: Apoplexy, 1; asphyxia, 1; asthma, 2; burned or scalded, 1; casualties, 2; cancer, 2; consumption, 25; convulsions, 9; croup or hives, 2; delirium tremens, 4; diarrhœa, 2; dropsy, 2; dropsy in the head, 5; dropsy in the chest, 1; dysentery, 1; erysipelas, 2; fever, scarlet, 7; do. typhoid, 1; do. puerperal, 3; do. bilious, 2; inflammation, 2; do. of brain, 10; do. of chest, 2; do. of lungs, 22; marasmus, 7; malformation, 1; measles, 5; organic disease of heart, 1; rheumatism, 2; suicide, 1; sprue, 1; small pox, 4; scrofula, 3; whooping cough, 1.

Ages—Of 1 year and under, 28; between 1 and 2, 19; 2 and 5, 23; 5 and 10, 2; 10 and 20, 2; 20 and 30, 21; 30 and 40, 14; 40 and 50, 13; 50 and 60, 4; 60 and 70, 8; 70 and 80, 1.

39 men—21 women—46 boys—29 girls. Total, 135.

### HEALTH OF THE CITY.

INTERMENTS *in the City and Liberties of Philadelphia, from the* 13*th to the* 20*th of March*, 1841.

| Diseases. | Adults. | Children. |
|---|---|---|
| Apoplexy, | 1 | 0 |
| Cachexia, | 0 | 1 |
| Cancer of stom'h, | 1 | 0 |
| —— womb, | 1 | 0 |
| Croup, | 0 | 2 |
| Cong'n of brain, | 0 | 1 |
| Consumption of the lungs, | 21 | 4 |
| Convulsions, | 0 | 8 |
| Cyanosis, | 0 | 1 |
| Cynanche tonsillaris, | 0 | 1 |
| Diarrhœa, | 1 | 0 |
| Dropsy, abdominal, | 0 | 1 |
| —— head, | 0 | 5 |
| —— breast, | 1 | 0 |
| Dis'se of the brain, | 1 | 0 |
| —— bladder, | 1 | 0 |
| —— hip-joint, | 0 | 1 |
| —— liver, | 1 | 0 |
| Dysentery, | 1 | 0 |
| Debility, | 1 | 1 |
| Enlargement of the heart, | 2 | 0 |
| Fever, scarlet, | 0 | 3 |
| Inflammation of the brain, | 2 | 2 |
| —— bronchi, | 0 | 10 |
| —— lungs, | 0 | 5 |
| —— breast, | 0 | 2 |
| —— bowels, | 1 | 1 |
| —— liver, | 0 | 1 |
| —— throat, | 0 | 1 |
| *Carried forward*, | 36 | 51 |

| Diseases. | Adults. | Children. |
|---|---|---|
| *Brought forward*, | 36 | 51 |
| Inflammation of the heart, | 0 | 1 |
| Intemperance and exposure, | 1 | 0 |
| Inanition, | 0 | 1 |
| Influenza, | 0 | 1 |
| Marasmus, | 0 | 1 |
| Malformation of heart, | 0 | 1 |
| Old age, | 1 | 0 |
| Palsy, | 1 | 0 |
| Pleurisy, | 1 | 0 |
| Small pox, | 2 | 6 |
| Still-born, | 0 | 10 |
| Suicide, | 1 | 0 |
| Total, 115— | 43 | 72 |

| Of the above, there were | |
|---|---|
| under 1 year | 36 |
| From 1 to 2 | 15 |
| 2 to 5 | 15 |
| 5 to 10 | 1 |
| 10 to 15 | 1 |
| 15 to 20 | 4 |
| 20 to 30 | 9 |
| 30 to 40 | 10 |
| 40 to 50 | 9 |
| 50 to 60 | 4 |
| 60 to 70 | 4 |
| 70 to 80 | 4 |
| 80 to 90 | 2 |
| 90 to 100 | 1 |
| Total, | 115 |

In the above are included 14 people of colour, 8 interments from the alms-house, and 1 from the country.

